# Focus on psychosocial risks and challenges in occupational medicine

**DOI:** 10.47626/1679-4435-2025-234

**Published:** 2026-01-05

**Authors:** Francisco Cortes Fernandes

**Affiliations:** 1 President of the National Association of Occupational Medicine, Executive Editor of the Revista Brasileira de Medicina do Trabalho

Dear readers,

It is with great satisfaction and gratitude to the authors that we conclude volume 23 of 2025 of the Revista Brasileira de Medicina do Trabalho (RBMT).

The diversity of topics presented and discussed in original research articles, reviews, opinion articles, and experience reports in the field of occupational medicine and workers’ health reinforces RBMT’s commitment and scientific relevance within the interdisciplinary scope of themes that address work as a key social determinant in the health-disease process of both formal and informal workers.

Work as a promoter of health has increasingly faced challenges related to the growing precariousness of working conditions, further exacerbated by information technologies that have intensified work processes and allowed new forms of work organization, such as telework and platform work. In this context, workers’ stress and suffering are reflected in the steep rise in mental disorders.

Considering the context of contemporary work environments, the first issue of this RBMT volume focused on articles addressing the role of psychosocial risk factors at work (PRFW) in the development of illness, while also publishing studies on traditional occupational risks, including work-related accidents and occupational diseases.

Following this direction, the final issue features articles on PRFW, workplace violence, mental disorders, suicidal behavior, burnout, presenteeism, accidents involving biological materials, mesothelioma, diabetes and work, as well as the impact of climate change on workers’ productivity.

We extend our sincere thanks to the journal’s editors for their efforts to meet SciELO’s requirements, as well as to the members of the editorial board and the peer reviewers, whose contributions have helped raise the technical and scientific standards of RBMT.

In accordance with SciELO’s recommendations, as of 2026, RBMT will discontinue its issue-based publication model (four issues per year) and will adopt continuous article publication on its website. At the end of the year, corresponding to volume 24, a single editorial closing the collection will be published.

